# Exploration of Nursing Care for Individuals With Bipolar Disorder in a Manic Episode: A Qualitative Study

**DOI:** 10.7759/cureus.63150

**Published:** 2024-06-25

**Authors:** Evangelos C Fradelos, Konstantina Gkatzogia, Aikaterini Toska, Maria Saridi, Ioanna Dimitriadou, Marianna Mantzorou, Afroditi Zartaloudi

**Affiliations:** 1 Laboratory of Clinical Nursing/Department of Nursing, University of Thessaly, Larissa, GRC; 2 Laboratory of Clinical Nursing/Department of Nursing, Health Center of Veroia, Veroia, GRC; 3 Department of Nursing, University of West Attica, Athens, GRC

**Keywords:** nursing intervention, nursing care, manic episode, mania, bipolar disorder

## Abstract

Background: Bipolar disorder is a mental illness that is chronic and has frequent relapses.

Objectives: The purpose of the research was to study the nursing care of patients with bipolar disorder in the mania phase.

Methods: A qualitative study was employed in this study. The sample consisted of 10 nurses working in psychiatric clinics and data were collected through semi-structured interviews. Thematic analysis was applied for analysing the data.

Results: Of the 10 participants, 70% were female and 30% were male. The mean age was 48.7 years. All participants were registered nurses and most of them held a Master of Science degree. Their work experience ranged from 10 to 30 years. Three main themes emerged when analysing the data obtained from the interviews with the nurses, those themes were a) Echoes of Vigilance: Navigating the journey, b) Amidst the Tempest: Attending to the Patients’ Complex Needs, and c) Restoring Balance: The Nurturing Hands of Bipolar Nursing Care, each of which could be divided into several sub-themes.

Conclusions: Nursing care plays an important role in symptom improvement and disease control by providing patient support, managing pharmacotherapy, preventing suicidality, and educating patients about the disease and self-management strategies.

## Introduction

Bipolar disorder is a chronic mental illness characterized by frequent relapses and emotional disturbances [1]. Bipolar disorder causes extreme mood swings, belligerent behaviour and disturbances in one’s thoughts and actions. Another characteristic of bipolar disorder is the alternation between the phases of depression and mania. Between phases, patients may experience their normal mood (normothymia) [2]. It should be mentioned that it is normal for a person to have shifts in mood. However, with bipolar disorder, these alternations appear with extreme characteristics and vary in duration and intensity. For example, joy changes to manic symptoms and sadness changes to depressive symptoms [3].

There are three types of bipolar disorder. All three types involve distinct changes in mood, energy and activity levels. These moods range from periods of extreme joy, excitement, irritability or energetic behaviour (known as manic episodes) to sad, indifferent or hopeless periods (known as depressive episodes). Less severe manic episodes are known as hypomanic episodes [4]. Type I bipolar disorder is defined by manic episodes that last at least seven days (almost every day for most of the day) or by manic symptoms that are so severe that the person needs immediate medical attention. Usually, depressive episodes also occur and last for at least two weeks. It is also possible to have depressive episodes with mixed features (with depressive symptoms and manic symptoms at the same time). Type II bipolar disorder is defined by a pattern of depressive and hypomanic episodes. Finally, cyclothymia is defined by recurrent hypomanic and depressive symptoms that are not severe enough or do not last long enough to qualify as hypomanic or depressive episodes [5]. Symptoms of mania, as described by Goodwin and Jamison, include excessive confidence and self-esteem, excessive optimism, excitement and euphoria, hyperactivity, hallucinations and delusions, aggression and irritability, elevated sexual desire, decreased need for sleep, lack of impulse control with inappropriate behaviours and incomprehensible and disorganised speech [4].

Nurses play an important role in the management and treatment of bipolar disorder, especially in its acute manic state. Nurses are facing many challenges while caring for patients suffering from bipolar disorder. A nurse is a member of the healthcare team who has frequent interactions with patients while caring for them. In addition, they are facing many difficulties in managing the emotions, thoughts and behaviours of patients with acute mania. At the same time, caring for patients in a manic state can cause various emotions such as joy, sadness, and anger. The first step a nurse should take when he or she has a patient with bipolar disorder in a manic phase is to perform a nursing assessment. The assessment focuses on the patient’s symptoms and particularly on thought process/perception, agency, volition/behaviour, emotion, and communication. The nurse also assesses whether the patient exhibits aggression towards themselves or others [6]. Nursing interventions for manic patients, as described by Goodwin & Jamison, include reducing environmental stimuli and creating a safe environment free of dangerous objects. At the start of the intervention, the patient is verbally guided and transferred to a quiet room. The patient may be restrained according to the hospital’s protocols. The situation will be explained to them in such a scenario. Nurses must avoid arguments and delays in granting the patient’s requests, as these can lead to aggression. Discussions of alternative problem-solving strategies take place when the patient is not agitated, while therapeutic communication is provided with simple and clear suggestions. Encouraging patients to discuss what triggers them, as well as rewarding them for effectively dealing with stressful events without violence and aggression, is important. Protocols for dealing with bipolar patients also include administering medications and monitoring their effectiveness and potential side effects, as well as working with other members of the multidisciplinary team [4].

The goals of treating an acute manic episode are to relieve symptoms and return to normal levels of psychosocial functioning. Achieving rapid control of agitation, aggression and impulsiveness is particularly important to ensure the safety of patients and those around them. Sometimes, due to a poor understanding of the disease and often problematic (involuntary) behaviour, hospitalisation is required to initiate effective treatment. Intensive nursing care is then required, along with pharmacotherapy [7].

There is a lack of evidence-based knowledge in the area of nursing care for patients in a manic state. Blegen et al. conducted a qualitative study to gain insight into nursing staff’s perceptions of patients experiencing a manic episode and how these perceptions influenced nursing care. Interviews with six nurses highlighted the need to understand each patient and their changing needs, as well as the need for nurses to work together as a team. It was also important that they had knowledge of the mania, maintained contact with the patient, remained clear about permissions and ward rules and maintained the measures needed to guarantee safety [8]. Hem et al. conducted a qualitative ethnographic study. They stated that boundary setting is a difficult task in nursing care for patients with mania because patients’ personal boundaries are sometimes overstepped [9]. This was also confirmed by the subsequent research of Daggenvoorde et al. [10]. Also, Goossens et al. showed that feeling safe, and respected and clear communication were vital treatment goals for patients [11].

Although nurses emphasise the importance of nursing care during manic episodes in bipolar disorder [8-11], several barriers to practice and care planning, such as inadequate time, understaffing, lack of specialised training, inconsistent documentation and lack of bipolar disorder-related knowledge, have been documented [12-14]. Despite the fact that nursing care for major depressive [15] disorder and other disorders [16] has been documented among Greek nurses, their views on providing care for bipolar disorder are under investigation.

Thus, this study aims to explore nurses' views regarding the provided care in patients with bipolar disorder when they have been hospitalized during a manic episode. The research questions include the first thoughts and feelings of nurses when they receive such patients, the main problems and the most important needs of the patients, the desired outcomes of their care and the proposed interventions to improve the care provided by and create a mentally healthy environment for the nurses.

## Materials and methods

In this study, we employed a qualitative design to achieve a detailed understanding of the nursing care provided to patients with bipolar disorder in the manic phase. A qualitative research methodology is considered the most suitable for this topic because standardising questions is not feasible or expected to yield precise results. Utilising qualitative techniques, such as observation and interviews, can provide answers to personal or sensitive questions.

Data were collected through semi-structured interviews. The demographic characteristics of the nurses were recorded, after which five open-ended questions regarding nursing care for patients with bipolar disorder in the manic phase were asked. Each interview lasted approximately 30-40 minutes. Subsequently, the interviews were recorded, transcribed and thematically analysed. The analysis was conducted in six stages: (1) transcribing and reading data and recording initial thoughts, (2) initial coding and systematic collection of relevant data for each concept across the entire data set, (3) searching for themes, (4) examining themes and checking their relevance to the extracted concepts and the entire data set, (5) analysis, improvement and naming of each section and (6) selection of findings [17].

The study was conducted in accordance with the Declaration of Helsinki and approved by the Ethics Committee of Department of Nursing Number: 357b 21/02/2022. The nurses were briefed on the research purpose and assured of their anonymity throughout the study. They were also informed that they could drop out of the research at any time if they wished to do so.

The research sample consisted of 10 nurses working in psychiatric clinics-three males and seven females. They were all registered nurses (RN) between 40 and 58 years old. Seven nurses held postgraduate degrees. All nurses collaborated with psychologists, psychiatrists, social workers and occupational therapists. Six nurses worked in State Psychiatric Hospitals in Greece and four worked in Psychiatric Clinics of Public Hospitals in Greece. Detailed samples are presented in Table 1.

**Table 1:** Sample Characteristics.

Participant	Gender	Age	Highest Education	Working Setting
Registered Nurse 1	Female	45	MSc	Psychiatric Hospital
Registered Nurse 2	Female	47	MSc	General Hospital – Acute Psychiatric Department
Registered Nurse 3	Female	58	MSc	Psychiatric Hospital
Registered Nurse 4	Male	40	Specialization	General Hospital – Acute Psychiatric Department
Registered Nurse 5	Male	45	MSc	Psychiatric Hospital
Registered Nurse 6	Male	57	Specialization	General Hospital – Acute Psychiatric Department
Registered Nurse 7	Female	54	Specialization	Psychiatric Hospital
Registered Nurse 8	Female	42	MSc	Psychiatric Hospital
Registered Nurse 9	Female	51	MSc	Psychiatric Hospital
Registered Nurse 10	Female	45	MSc	General Hospital – Acute Psychiatric Department

In order to achieve the validity of the results after the generation of themes and sub-themes, the results were reviewed by two members of the research team who agreed that the suggested themes and sub-themes are supported by the data collected.

## Results

Of the 10 participants, 70% were female and 30% were male. The mean age was 48.7 years. All participants were RNs and most of them held a master’s in science degree. Their work experience ranged from 10 to 30 years. Three main themes emerged when analysing the data obtained from the interviews with the nurses, each of which could be divided into several sub-themes. The findings regarding these themes and sub-themes are presented in Table 2.

**Table 2:** Themes and Sub-themes Emerged From the Analysis and Quoting.

Themes	Sub-themes	Quotations
A. Echoes of Vigilance: Navigating the journey	Being Vigilant	RN3 “Because we don't know how long it's been since the onset of symptoms, how neglected he is, how aggressive he is. … We make sure to be placed in a single room during manic episode…so the patient can eat and rest.''
	Maintain Safe Environment	RN 7: “When they come to your department, they create the feeling of vigilance. They are hyperactive, they are patients who overestimate their strength, so we have a great responsibility, in terms of medication, diet, sleep, in not getting involved in fuss with other patients.”
	Building Trust	RN 4: “Trust is one of the most important things that nurses must build. When they (patients) come to the department, they are tense, talkative, hyperactive, distracted... When in a manic episode, some people are dangerous because they overestimate their powers. They cooperate more easily in their diet than in their medication. So, creating a trustful relationship is a key component in nursing care for those patients.”
	Nursing Evaluation	RN 10: “The domains that should be evaluated is including physical assessment, these patients do not get tired. We will evaluate the thought process, the perception, if he is distracted, if he has hallucinations, delusions of grandeur. We will also observe his hygiene if he is clean, we will assess communication if he has disorganised speech. Then, we should assess whether he tends to harm himself or others.”
B. Amidst the Tempest: Attending to the Patients’ Complex Needs	Sleep Disturbance	RN 3: “The first goal is to get out of the manic phase… to help him sleep and rest and take care of him.”
	Hyperactivity and garrulity	RN 3 mentioned the following: “You immediately understand that these people need hospitalisation. In the rage phase, it is difficult to handle. We have to be careful. These patients are tense when they come, they talk all the time, they don’t sit, they walk all the time.”
	Lack of Insight	RN 1: “Some patients refuse to collaborate… they are not fully aware of their condition and their state…”
	Lack of Social Support and Impaired Social Relationships	RN 10: “During recovery, we try to help patients to improve their social life and reobtain their social skills.”
	Mood Swings	RN 9: “Their emotion is swift and we must help them balance it , to not be aggressive, not to spend money and stay out of trouble.”
	Non-Adherence in Treatment	RN 4: “They are not so cooperative in medications… as opposed to other caring activities”
	Need of Support	RN 2, “Some patients don’t have a supportive environment; they have a sick family environment. He may have difficulties that have not been dealt with. He may also have some traumatic events.”
C. Restoring Balance: The Nurturing Hands of Bipolar Nursing Care	Family Education and Training	RN 2: “What we want is the fewest possible relapses, and this can happen by providing people with psychoeducational interventions, adjuncts to pharmacotherapy, fewer hospitalisations, psychoeducation in the family, creating very good structures, strengthening friendly environments. To educate the patient from all specialties related to mental health. Education should also be done in the family. Bipolar patients need special handling”.
	Personalized Nursing Care	RN 3: “Each patient requires a specialised approach… has its own needs and many times requires a different approach…”
	Setting Boundaries	RN 9: “The approach must be very careful… Should avoid conflict at all times…boundaries are needed and you shouldn’t be carried away by emotion.”
	Promoting Medication Adherence	RN 8: “Main goal is medication adherence, is that best way to prevent relapse”
	Emotion Regulation and Relapse Prevention	RN 2 … “Patients should learn to manage their emotional pressures, fatigue, increased stress levels.”
	Building Therapeutic Relationship	RN 6 stated, “We must create a protected and supportive cycle around the patient to promote relationship building.”
	Nutrition Management	RN 7 “Patients usually don’t have a daily routine and their nutrition is not healthy and balanced. We have a great responsibility to maintain their health, medication administration and nutrition management.”
	Promoting Selfcare and Independent Living	RN 3 “‘our fear is to watch him go into depression”. In other words, the nurses emphasised helping patients return to normal.
	Quality οf Life	RN 2, “Rehabilitation is very crucial…to help a patient return to his social environment and to retain abilities once lost.”

Theme A: echoes of vigilance: navigating the journey

Many nurses reported that their thoughts and emotion were mixed during their first contact with the patients.

Being Vigilant

Being vigilant and attentive to the needs and behaviours of patients with bipolar disorder, nurses stated that they were alert, stressed and felt a sense of responsibility when caring for patients with bipolar disorder in manic episodes.

RN 8: “*When you see the patient coming, you are on high alert and you feel that everything must be done quickly in order to immediately reduce the manic symptoms. We worry about these patients because we see that they are confused... besides, they are usually accompanied by police officers, so this is very heavy for their psyche. I definitely feel both stress and worry because I feel like I have a responsibility… a big responsibility… to help this man get back to normal levels.”*

Another issue that emerged was the uncertainty that nurses experience when they care for bipolar patients during the first hours of admission.

Maintaining a Safe Environment

Nurses ensure the physical and emotional safety of patients. Their priority is the safety of the patient, ensuring that he or she does not harm himself or those around him or her. Another priority is ensuring that the patient sleeps, eats, and starts medication quickly. Achieving rapid control of agitation, aggression, and impulsivity is particularly important to ensure the safety of patients and those around them.

RN 4: *“The first goal is his safety, because it is difficult to deal with him in the acute manic phase, especially if you do not know him. ………patient who has come again, maybe we know how dangerous he is and we have a previous experience on how he behaves during his hospitalisation”.*

Building Trust

Since nurses reported not knowing what to expect, the importance of having previous experience with patients and building trust was highlighted. Additionally, according to the participants, establishing a trusting relationship between nurses and patients facilitated effective care. As RN 4 stated *“Trust is one of the most important things that nurses must build".*

Nursing Evaluation

Participants stated that regular assessment and evaluation of patients’ needs and progress were their main nursing tasks when caring for bipolar patients. RN 10: *“The domains that should be evaluated is including physical assessment, these patients do not get tired. We will evaluate the thought process, the perception, if he is distracted, if he has hallucinations, delusions of grandeur". *

Theme B: amidst the tempest: attending to the patients’ complex needs

According to nurses, patients with bipolar disorder often experience a range of symptoms and needs that require careful attention and management.

Sleep disturbance is a common issue, necessitating interventions to address disruptions in sleep patterns and to promote better quality rest. According to the nurses, patients with mania do not sleep. Patients tell them that they are not tired, that they do not need to sleep, and that sleep is a waste of time. These energetic patients seem to always be busy with some activity and want things done immediately. However, the patients’ behaviours are chaotic, exhausting, and lack structure. As RN 3 stated: *“The first goal is to get out of the manic phase… to help him sleep and rest and take care of him.” *

Hyperactivity and garrulity are often observed during manic episodes. Containing this requires strategies to manage increased activity levels and restlessness. Also, nurses report that these patients often talk nonstop and do not tolerate any interruption or contradiction. When this happens, they usually respond with “Yes, but...” Boundaries are not accepted and patients are restless, talk loudly, and their agitation often leads to verbal or non-verbal aggression. There is a loss of formality. Patients often disagree with those around them (e.g., family, care professionals, other patients). This behaviour continues around the clock, which may disturb others. Patients are often angry, and this anger can quickly flare up. RN 3 mentioned the following: *“You immediately understand that these people need hospitalisation. In the rage phase, it is difficult to handle. We have to be careful. These patients are tense when they come, they talk all the time, they don’t sit, they walk all the time.”*

RN 5 mentioned the following: *“We make sure that he does not get involved in fights with other patients because in the mania phase they disturb the others with the intense speaking mood they have. In the rage phase, they demand things they want. The manic patient stands out; he does everything in excess in a few words. Their appearance is pronounced mainly in women. They dye brightly, wear many accessories. They are restless, they walk constantly, they share their things, they have ideas of grandeur.”*

Lack of insight into their condition is another challenge, highlighting the importance of assisting patients in recognising and understanding their illness and its impact on their lives. Furthermore, nurses reported that patients hospitalised with mania often claimed to not be sick. As RN 1 stated *“Some patients refuse to collaborate… they are not fully aware of their condition and their state…”* Patients overestimate themselves and show little or no awareness of their illness and insight into their behaviour and its consequences. Patients often refuse to take their medication and don’t want to discuss why. Patients do what they want, and they insist on doing it.

Lack of social support and impaired social relationships are other challenges. Nurses highlighted the social impact that this disease can have on patients’ lives. Addressing issues related to a lack of social support and impaired social relationships is crucial for patients’ well-being. According to nurses, the lack of a support network is one of the main problems that patients face. Furthermore, unhealthy family environments and traumatic experiences are important problems. Problems in personal relationships were reported as a result of the illness, including changing relationship dynamics, increased tension in relationships, and loss of trust.

RN 7: “*Among others, we must help in order the relationship with family and friends is re-established.”*

Mood swings, a characteristic of bipolar disorder, require ongoing management to navigate fluctuations in mood and emotional states. Changes in patient behaviour reflect mood instability and are reported by nurses to be a major problem. Mania causes changes in sleep patterns, energy levels, activity levels, and overloaded schedules. Nurses report paying close attention to nonverbal signs and symptoms. They look out for physical and mental signals. They may also consider the patient’s hygiene, dressing, and speech patterns when looking for potential signs of an incoming manic episode.

RN 9: *“During spring time, their emotions are more intense … they’re more euphoric …. Maybe due to vitamin D … Who knows…”*

RN 2: *“we must try to balance their emotions. That’s why we should form an alliance with them….”*

Non-adherence to treatment plans can pose significant obstacles to recovery, underscoring the need for interventions aimed at addressing patients’ reluctance or refusal to follow prescribed therapies.

RN 9:* “It is important that they understand the medication, because they often resist taking their medication.*

Need of Support

Comprehensive support and resources are essential to help patients cope effectively with their condition and improve their quality of life. According to RN 9, “*[the] patient must understand that you really care about him. Our approach should always be consultative and not prescriptive. Also, the patient should have the opportunity to talk about his problems…’’.*

RN 10 stated, *“patients are in need of our support, and a nurse maintains a supportive attitude, but our communication with the patient should be realistic.”*

Theme C: restoring balance: the nurturing hands of bipolar nursing care

According to the participants, nursing care focuses on many areas to achieve the desired outcomes for patients with bipolar disorder. Through educational programs for the family, a better understanding of the disease and patient support is sought. Individualised nursing care is tailored to the needs and preferences of each patient, while maintaining boundaries is an important measure in maintaining a healthy therapeutic relationship. Promoting medication adherence is critical to successful disease management, while emotional regulation and relapse prevention are important goals. Creating a therapeutic relationship is based on mutual trust and communication, while creating a routine helps with stability and self-management. Managing nutrition and promoting self-care and independent living help improve patients’ quality of life.

Family Education, Information Sharing and Training

According to nurses, providing information to patients and their families is an important intervention. Providing clear information about the ward’s rules upon admission-both verbally and in writing-and repeating those rules when the patient breaks them are important. Information about treatment and psychoeducation about the illness should be provided on a regular basis. As RN 2 stated “*psychoeducation in the family, creating very good structures, strengthening friendly environments. To educate the patient from all specialties related to mental health. Education should also be done in the family. Bipolar patients need special handling”*.

Personalized Nursing Care

Each patient requires an individual nursing approach that is adapted to his or her needs. It is important that the nurse has a supportive attitude and approach to the patient, being present and approachable. Nurses must show personal attention and involvement with the patient and cooperate with the patient’s family. It is important for the nurse to connect with the patient and stay connected to encourage the desired behaviour.

RN 5: “*How many days is in this condition, if he has eaten, if he has slept. In other words, we take care of their specific needs that exhibiting and that he starts his medication immediately.*”

Setting Boundaries

The participants suggested that setting boundaries is a key intervention in the treatment of patients with mania. Although nurses must clearly communicate which behaviours are desirable and which are not, it is important to focus on affirming the desired behaviour rather than addressing the undesirable behaviour. Excessive use of boundaries and rules can escalate undesirable behaviours. When the nurse sets limits with persuasion and humour and communicates correctly with the patient, the limits are more easily accepted. Whether the patient respects the boundaries depends not only on the patient’s psychiatric condition, but also on the attitude and approach of the nurse.

RN 8: “*First, you must think the way that you approach the patient. It is very difficult to set boundaries and boundaries are really needed during that hospitalisation of bipolar patients*.”

Promoting Medication Adherence

Medication administration and adherence are vital to the remission of manic symptoms. Often, patients refuse to take their prescribed medications. Nurses must spend time motivating patients to take medications. Responding to past experiences and repeating information about a medication’s effects and possible side effects can prove sufficiently motivating. The nurse should check that the patient has actually taken his medications and observe their effectiveness and possible side effects. If the patient refuses to take medication, the nurse should find out why the patient is doing so.

RN 2:*“It is important that they understand the medication, because they often resist taking their medication”.*

If the patient has questions about the medication, the nurse should let the patient know they can talk to a psychiatrist. Also, as RN 6 mentioned that *“‘you have to ask the patient about the side effects because otherwise he usually doesn’t say it on his own.”*

Emotional Regulation and Relapse Prevention

According to the participants, the emotional regulation of bipolar patients is essential to managing their symptoms and maintaining stability. Through emotional regulation, nurses can help patients cope with mood swings and prevent worsening symptoms. Additionally, emotional regulation helps create a supportive environment that promotes patient wellness and recovery.

RN 7 “*A patient must return in an emotionally state where he can manage himself…”*

Building a Therapeutic Relationship

Building therapeutic relationships is critical in caring for bipolar patients; patients feel comfortable and safe to express their concerns and needs to the nursing staff. This allows nurses to better understand individual patient needs and tailor care accordingly. Furthermore, trust contributes to the formation of a positive communication environment, which enhances mutual understanding and cooperation between nurses and patients. Consequently, establishing a trusting relationship helps achieve positive treatment outcomes and cope with the fluctuations associated with bipolar disorder.

RN 7 stated,* “You get great satisfaction because you take care of people who have no others. We celebrate Christmas and Easter together. Mental illnesses last a long time, and as a result, relatives get tired and leave them. We bond with these patients.”*

Nutrition Management

Nurses help the patient to follow a healthy and balanced diet, to find the skills that he has lost, and to observe if he has a communication disorder. The nurse maintains a supportive attitude, and as RN 7 mentioned, *“Patients usually don’t have a daily routine and their nutrition is not healthy and balanced. We have a great responsibility to maintain their health, medication administration and nutrition management.”*

Promoting Self-Care and Independent Living

Self-care and independent living constitute a desired outcome for nursing care. Most of the nurses made self-management assessments of patients’ abilities to recognise the early signs or symptoms of a manic episode and their abilities to deal with these early warning signs. Another reported indicator is the patient’s ability to recognise their vulnerabilities and take the necessary actions to address those vulnerabilities. When patients come out of the manic phase, the nurses help them to become independent, take care of themselves, take their medication, leave the hospital, and regularly undergo testing. RN 7 said, *“To return the patient emotionally to a situation where he can manage himself. To take his medicines correctly, to eat properly, to do his tests, not to be dangerous to others, not to overestimate their powers, not to get involved in fuss. To take care of himself to be clean, to have good relations with his family, relatives and friends."*

Quality of Life

Another desired outcome is to improve patients’ quality of life. This is defined by nurses as a patient’s ability to fulfill various social roles. This ability can be assessed indirectly by monitoring the patient’s level of satisfaction with daily activities, the patient’s self-esteem, and the patient’s level of satisfaction. Both acceptance of their chronic illness and understanding the illness are also recognised as desirable outcomes. These outcomes are usually assessed in terms of self-management skills, the patient’s ability to recognize problems/vulnerabilities and the patient's ability to cope with the consequences of such a situation and thereby avoid episodes of (hypo)mania and depression as much as possible.

RN 10 stated, *“After the manic episode, they should return to their life and occupation to be active members of the communities and to live in fulfilment.”*

## Discussion

This study examined the nursing care of patients in the manic phase due to bipolar disorder. Nurses treat such patients with care and vigilance, focusing on their safety and prompt initiation of medication. They deal with various problems that patients may face, such as a lack of a support network and problems with personal relationships. They also focus on promoting patient self-management and encouraging them to lead healthy lives. Communication with the patient is central, with nurses using clear and respectful communication. Overall, care is focused on the patient’s safety, health and well-being. Nurses of the study considered mood stability as a major desired outcome for patients with manic bipolar disorder because it is essential for daily life. Nurses also mentioned that they must collaborate with a multidisciplinary team, educate families and collaborate with the patients’ caregivers to improve care. Additionally, they must ensure that the environment to which patients return is appropriate. Finding work and creative employment for patients can help prevent relapses. Communication with patients must be tailored to their needs, and boundary setting was regarded by nurses as an important intervention.

These findings are consistent with those of similar studies [9-11,13,18]. The first step a nurse must take when caring for a patient with bipolar disorder in a manic phase is to conduct a nursing assessment. The assessment focuses on the patient’s symptoms, particularly on thought processes/perception, organicity, will/behaviour, emotion and communication. The nurse also evaluates whether the patient exhibits aggression towards themselves or others [19]. The goals of treating an acute manic episode are symptom relief and returning to the usual psychosocial functionality. Achieving rapid control of agitation, aggression and impulsiveness is particularly important to ensure the safety of patients and those around them. Sometimes, due to poor insight into the illness and often problematic behaviour, hospitalisation is required to initiate effective treatment. Subsequently, intensive nursing care, along with pharmacotherapy, is necessary [11].

The nurses of the study declared that medication administration and adherence are vital to the remission of manic symptoms. Often, patients refuse to take their prescribed medications. Nonadherence to treatment is one of the most significant problems, as strong evidence indicates that nonadherence to treatment among patients with bipolar disorder is the most common cause of episode recurrence. The participating nurses mentioned that if the patient refuses to take medication, the nurse should find out why the patient is doing so. Cognitive impairment in the form of decision-making problems, planning, verbal memory, working memory, attention/cognitive control, and information processing may affect adherence to pharmacological treatment [13].

The participants suggested that setting boundaries is a key intervention in the treatment of patients with mania. Acceptance of the boundaries set by nurses for patients appears to be associated with two factors: the nurse’s attitude and the patient’s mood. The nurse’s communication and negotiation skills are important [20,21]. Nurses must be creative to find a balance between being clear and strict about rules and being flexible in responding to individual patients’ needs [10]. Creativity and flexibility are important nursing skills in providing pharmacological treatment to patients. Although motivational interviewing is known to show promising results in improving adherence to pharmacological treatment in patients with bipolar disorder [22,23], it is unknown whether this intervention is applicable to patients during manic episodes.

According to the nurses of the sample, patients with bipolar disorder often experience a range of symptoms and needs that require careful attention and management. The main problems for patients include disrupted day-night rhythm, agitation, lack of illness insight, verbal aggression and excessive physical activity. On the other hand, the main nursing interventions include setting boundaries, motivation for medication adherence, adequate medication administration, structuring day-night rhythm and supportive communication. The most frequently mentioned intervention is boundary setting, which requires building and maintaining a connection with the patient. Adjusting communication for each patient and his/her specific symptoms is considered an excellent starting point for such a connection. At the very least, nurses should communicate which behaviours are acceptable (and expected) and which are not. Additionally, as part of their daily work, nursing teams care for a group of patients and must ensure the safety and well-being of all group members and staff. It is also recommended to reflect on nurses’ awareness of potentially conflicting rules, beliefs and emotions upon which their interactions with patients and their behaviour are based [15].

Nurses of the sample stressed the importance of having a supportive attitude and approach to the patient, being present and approachable. Rantala and colleagues argue that nurses’ ability to create a relaxing atmosphere is important because doing so can lead patients who are insecure to feel strong enough to participate in decision-making [24]. Devoting time to listening to patients-which includes empathy, silence, attention to both verbal and non-verbal communication and the ability not to criticise and to accept-has always been considered a critical component of nursing care [25]. Listening to the patient is important to assess the patient’s mental health status and to create order in the patient’s perceptible existential chaos. Since nurses reported not knowing what to expect, the importance of having previous experience with patients and building trust was highlighted. However, a nurse not knowing what to say to a patient with a mental illness could negatively impact the patient and reduce his/her trust in verbal communication [26].

This research has some limitations that need to be noted. The first is the qualitative study design and the second is the small sample size. purposeful sample and some biases of the interviewers should be taken into account. Neither of these factors allowed generalisation of the results.

## Conclusions

Bipolar disorder is a complex mental problem that affects the lives of many people, causing episodes of mania and depression that disrupt their daily lives. The treatment of bipolar disorder is a long-term and complex process, requiring a combination of medication and psychotherapeutic interventions. According to our results, caring for patients with bipolar disorder requires constant vigilance and building trust to facilitate medication management and support in their daily lives. Patients in a manic phase may exhibit hyperactivity, lack of awareness of their situation, and a need to restore sleep and balance. It is important to assess their mental and physical conditions while educating families and promoting a supportive environment to help reduce relapses and enhance their quality of life.

Nursing care plays an important role in symptom improvement and disease control by providing patient support, managing pharmacotherapy, preventing suicidality and educating patients about the disease and self-management strategies. By taking these initiatives, the nurse helps to create a safe and confidential environment, thereby helping patients to cope effectively with the disease and achieve long-term recovery.
